# A New Source and Large Quantity of Resveratrol in *Cratoxylum* Species and Their Activities on Normal Human and Cancer Cells

**DOI:** 10.3390/biology13060402

**Published:** 2024-06-01

**Authors:** Sanit Kaewdaungdee, Tankun Banlue, Napatsakon Youngsanbhu, Mallika Naeklang, Shiou Yih Lee, Arnold Ang, Runglawan Sudmoon, Tawatchai Tanee, Sakda Daduang, Arunrat Chaveerach

**Affiliations:** 1 Department of Biology, Faculty of Science, Khon Kaen University, Khon Kaen 40002, Thailand; sanit_k@kkumail.com (S.K.); tankun_b@kkumail.com (T.B.); immnapatsakon000@gmail.com (N.Y.); mallika.naeklang@kkumail.com (M.N.); 2 Faculty of Health and Life Sciences, INTI International University, Nilai 71800, Negeri Sembilan, Malaysia; shiouyih.lee@newinti.edu.my (S.Y.L.); i19017161@student.newinti.edu.my (A.A.); 3 Faculty of Law, Khon Kaen University, Khon Kaen 40002, Thailand; rungla@kku.ac.th; 4 Faculty of Environment and Resource Studies, Mahasarakham University, Maha Sarakham 44150, Thailand; tawatchai5@hotmail.com; 5 Division of Pharmacognosy and Toxicology, Faculty of Pharmaceutical Sciences, Khon Kaen University, Khon Kaen 40002, Thailand; sakdad@kku.ac.th

**Keywords:** *Cratoxylum*, resveratrol, α-amyrin, CHL-1, HCT-116, HepG2, PBMCs

## Abstract

**Simple Summary:**

We have made the first finding of resveratrol in the leaves of *C. sumatranum* and identified that all *Cratoxylum* extracts are toxic to cancer cell lines but are only slightly toxic to PBMCs at the cell level and are not harmful to DNA levels. All the studied extracts induced both apoptosis and necrosis in cancer cell lines, as evidenced by a significant decrease in DNA quantity in the S and G2-M phases. *C. sumatranum* extracts did not induce apoptosis and necrosis in PBMCs and showed a high percentage of cell viability, but the ethanolic extracts of the two subspecies of *C. formosum* induced apoptosis and necrosis in PBMCs. These data confirmed that the three studied *Cratoxylum* samples have inhibition activity on the growth of cancer cells and low toxicity to PBMCs. *C. sumatranum* showed better biological activity than *C. formosum* leaf extracts, which may be due to the combined activity of substances such as α-amyrin and resveratrol, which are proposed to be of a high quantity and similar function as anti-inflammatory substances. All studied samples deserve clinical trials for further application to plant-derived products, especially *C. sumatranum*. This knowledge renders the plants more useful for human health than commonly used as vegetables, cooking and traditional medicines. The plants can be used in other modified forms such as functional foods, supplements, drugs and nutraceuticals.

**Abstract:**

*Cratoxylum formosum* ssp. *formosum* (Cff), *C. formosum* ssp. *pruniflorum* (Cfp), and *C. sumatranum* (Cs) were investigated for phytochemical analysis. Toxicity testing, programmed cell death, and cell cycle arrest were tested on CHL-1, HCT-116, and HepG2 cancer cell lines, and human normal PBMCs. The results are revealed in the following order. The phytochemical percentages varied in each species, the quantity and concentration of α-amyrin and resveratrol were 0.038 mg/g and 0.955 mg/mL, and 0.064 mg/g and 0.640 mg/mL. The most studied *Cratoxylum* extracts showed IC_50_ values in PBMCs and cancer cell lines except for the hexane Cff and ethanol Cfp extracts. All studied extracts did not induce DNA breaks in PBMCs but caused significant DNA breaks in the cancer cell lines. All studied extracts induced both apoptosis and necrosis in cancer cell lines, and the DNA quantity in the S and G2-M phases decreased significantly but did not induce apoptosis and necrosis in PBMCs. Except for the ethanolic extracts of Cff and Cfp that induced PBMCs apoptosis and necrosis, these data confirmed that the three studied *Cratoxylum* samples have inhibiting properties for the growth of cancer cells and low toxicity to PBMCs. Cs showed more toxicity to cancer cell lines than Cf and cisplatin.

## 1. Introduction

The *Cratoxylum* species belonging to Hypericaceae represent very interesting plants as their young shoots, leaves and flowers have long been used for cooking, as a seasoning or the main ingredient in soup, or a vegetable side dish especially in northeastern Thailand. Six *Cratoxylum* species in Thailand have been found distributed throughout all regions namely *C. arborescens*, (vahl) Blume; *C. cochinchinense* (Lour.) Blume; *C. formosum* (Jack) Dyer, ssp *formosum*; *C. formosum* (Jack) Dyer, ssp *pruniflorum*; *C. maingayi* Dyer and *C. sumatranum* ssp. *neriifolium* Kurz [1,2]. Accordingly, Juanda et al. [3] reported that the *Cratoxylum* genus includes six accepted species distributed in Southeast Asia. The plants are being used in the traditional medicine system in Asian countries such as China, Indonesia, Malaysia, Thailand, and Vietnam due to their traditional uses, phytochemical compounds, and pharmacological activities. The major phytochemical compound discovered in all *Cratoxylum* species is xanthone, while the other compounds, anthraquinone, triterpenoid, steroid, flavonoid, phenolic acid, vismione, benzophenone, and tocotrienol, are found in various species. The medicinal properties of each plant depend on its chemical constituents, and many xanthones have been evaluated for their biological activities [4]. The extract of *Cratoxylum* species has a wide range of pharmacological activities such as cytotoxicity, antiplasmodial, antibacterial, antioxidant, antiulcer, α-glucosidase inhibitor, anti-inflammatory, and anti-cancer [3,5,6]. In 2023 [7], two xanthones, gerontoxanthone I and macluraxanthone, were isolated from the stem bark of *C. sumatranum*. Their structures were determined based on UV, HRESI-MS, 1D, and 2D NMR spectral data. The two substances showed high activity against T47D cells with an IC_50_ of 1.10 and 1.60 μg/mL, respectively [7]. Bok et al. [8] reviewed research into the *Cratoxylum* species and reported on their wide spectrum of phytoconstituents including xanthones, triterpenoids, flavonoids, and phenolic compounds. These compounds explain the plants’ significant pharmacological effects, such as their antibacterial, antifungal, antioxidant, antimalarial, anti-gastric ulcer, anti-HIV-1 reverse transcriptase, antidiabetic, and anti-cancer activities. These substances and pharmacological effects have strengthened the use of the *Cratoxylum* species as traditional medicinal plants and it is recognizes that they must be further developed and applied as selective therapeutic drugs for various ailments.

Currently, there is no recent research on the properties of this group of plants, from their known characteristics to the chemicals they contain, as gathered from the literature reviews. Huong et al. [9] studied the phytochemical and pharmacological properties and reported that xanthone derivatives, comprising 205 compounds, are the major secondary compounds (74%). These are followed by other chemical classes such as flavonoids, anthraquinones, triterpenoids, benzophenones, phytosterols, and tocopherols. The constituents of *Cratoxylum* possess a range of pharmacological activities, including antioxidant, antibacterial, anti-inflammatory, antidiabetic, antihypertensive, antimalarial, anti-viral, antiamoebic, protein tyrosine phosphatase 1B inhibitory, neuroprotective, hepatoprotective, and gastroprotective activities, and anti-cancer effects. Dai et al. [10] identified a new xanthone derivative, cratoxanthone, along with seven known biogenetically related xanthone derivatives in the phytochemistry of the stems of *Cratoxylum formosum* (Jack) Dyer subsp. pruniflorum (Kurz) Gogelin. The structures of the compounds were elucidated using comprehensive spectroscopic methods. These xanthones may play a role as chemotaxonomic markers for *C. formosum*.

Whilst *Cratoxylum* species are widely used in traditional medicines and have many pharmacological activities, some species however need further research regarding the mechanism of their pharmacological activities and chemical constituents. Therefore, this research selected three samples of two species *C. formosum* ssp. *formosum*, *C. formosum* ssp. *pruniflorum*, and *C. sumatranum*, for studying their biological activity including constituent phytochemicals, cytotoxicity, and genotoxicity via testing on PBMC and CHL-1, HCT-116, and HepG2 cell lines, programed cell death evaluation, and cell cycle arrest. These species were chosen due to their popularity for consumption, ease of sample collection, and preliminary screenings indicating notable pharmacological activities.

## 2. Materials and Methods

### 2.1. Plant Materials

The leaves of *Cratoxylum formosum* ssp. *formosum* (Jack) Dyer, *C. formosum* ssp. *pruniflorum* (Kurz) Gogel, and *C. sumatranum* (Jack) Blume were collected from public forest areas in the Udon Thani province, Thailand. The plants were identified by a proficient botanist, Prof. Arunrat Chaveerach, Ph.D. The voucher specimen numbers are A. Chaveerach 1089, A. Chaveerach 1090, and A. Chaveerach 1091. The sample leaves were cleaned and dried under room temperature conditions. The dried leaves were kept in a zip-lock bag containing silica gel beads to pull the moisture away.

### 2.2. Phytochemical Extraction

The dried *Cratoxylum* leaves were finely ground, and 25 g powder was macerated in 250 mL of ethanol and hexane separately at 25 °C for 72 h. The choice of solvents (ethanol and hexane) was justified by their ability to extract different types of compounds, their safety considerations, and the intended application of the extracts. After that, the extracts were filtrated through filter paper (Whatman no. 1). Five milliliters of each *Cratoxylum* leaf extracts were aliquoted into a new tube to further study the phytochemical composition. The solvents were eliminated from the remaining extracts by rotary evaporator (Rotavapor R-210; Buchi, Basel, Switzerland) at 800–1000 mbar, 600 rpm, and 42 °C for 2 h. Subsequently, the crude extracts were completely re-dissolved with 10% DMSO and kept at −20 °C for further studies; these solutions were kept as stock concentrations of the extracts. The % yield of each crude extract was calculated from [weight of extract after solvent elimination(g)/weight of plant sample(g)] × 100. 

### 2.3. Phytochemical Preparation for Phytochemical Composition and Identification Analysis

The aliquoted extracts were concentrated using a speed vacuum concentrator at 25 °C for 24 h. After that, the derived extracts were re-dissolved with 2 mL of the extracted solvent and centrifuged at 12,000 rpm for 3 min. The supernatant was collected. Then, 1 mL of each extract was fractionated with 1 mL of the mixed solvents between petroleum ether/diethyl ether (2:1 *v*/*v*). After precipitation, the clear solution was filtrated through a 0.45 µm syringe filter into a new tube for the phytochemical composition analysis.

### 2.4. Phytochemical Composition Analysis Using Gas Chromatography–Mass Spectrometry (GC-MS)

The prepared extract was subjected to GC-MS analysis using Agilent 5977B GC/MSD (Agilent Technologies, Inc., Santa Clara, CA, USA) equipped with HP-5MS UI column (30 m × 250 um × 0.5 um). Helium was used as the carrier gas at the constant flow rate of 1 min/mL. The oven temperature was slightly modified from Konappa et al. [11] and programmed at 50 °C (held for 3 min) to 280 °C at 10 °C/min for 10 min; the final temperature was raised up to 300 °C at 10 °C/min for 10 min. The injector temperature was constantly set at 250 °C. The injection volume was 1 µL with split mode at 10:1. The MS quadrupole and source temperature were programmed at 150 and 230 °C, while the mass ranged from 40 to 600 *m*/*z*. The compounds were identified using Agilent Mass Hunter Qualitative Analysis 10.0 which compared the mass spectra with the National Institute of Standards and Technology (NIST) library.

### 2.5. α-Amyrin Detection by Gas Chromatography–Frame Ionization Detector (GC-FID)

The prepared extracts were 2-fold serially diluted with the extracted solvent. The targeted compound was examined by GC using an 7890B GC system (Agilent Technologies, Inc., Santa Clara, CA, USA), equipped with flame ionization detector (FID). Helium was used as the carrier gas, at a flow rate of 1 mL/min. The injection temperature was 290 °C and detection temperature was 300 °C. The temperature program began at 200 °C and increased by 2 °C/min up to 300 °C, which was held for 20 min. The compounds were injected into a HP-5 capillary column (30.0 m × 0.32 mm i.d. × 0.25 μm film thickness). A split injection with a ratio of 1:10 was used. The 1 mg of α-amyrin standard was prepared and dissolved in 1 mL ethanol. The standard solution was 2-fold serially diluted for five levels (0.0625–1 mg/mL).

### 2.6. Resveratrol Investigation Using Reverse-Phase-High Performance Liquid Chromatography (HPLC)

The derived 10 g powder of each *Cratoxylum* species was macerated with 80% ethanol. All steps were performed according to the procedures in Section 2.2. Subsequently, the targeted compound was identified using reverse-phase HPLC (LC-20AD; Shimadzu, Kyoto, Japan) equipped with a quaternary pump, PAD (SPD-M20A) detector, and a C18 (Inertsil ODS-3) analytical column (4.6 mm × 250 mm, 5 micron particle size). The isocratic elution with a flow rate of 1.0 mL/min was used with the mobile phase of 25% acetonitrile and 1% phosphoric acid (10:90 *v*/*v*). The wavelength was set at 320 nm. The resveratrol (trans-3,4’,5-trihydroxystilbene) standard was prepared at 1 mg/mL in ethanol and 2-fold serially diluted for five levels.

### 2.7. Phytochemical Preparation for Toxicity Evaluation 

Stock concentrations of the extracts, as obtained in Section 2.2. were serially diluted 2-fold with sterile water to achieve eight different concentration levels. Consequently, the original 10% DMSO concentration was also proportionally diluted. After dilution, all solutions were filtrated through 0.45 µm syringe filter and stored at −20 °C to be employed as working concentration. The 10% DMSO was employed as a vehicle control. Furthermore, the cisplatin (Cas. no. 232120; Merck, Rahway, NJ, USA) was prepared for four concentration levels to be compared on efficiency with studied plant extracts.

### 2.8. Melanoma Cell (CHL-1), Colon Cancer Cell (HCT-116) and Hepatoblastoma Cell (HepG2) Preparation

The preparation steps followed the protocol of Arimilli et al. [12]. Briefly, the cells were thawed, washed, and then suspended in a low-glucose DMEM medium supplemented with 10% FBS, 100 µg/mL streptomycin, and 100 U/mL penicillin, at a concentration of 2 × 10^5^ cells/mL. For the experiment, the cell suspension was seeded into 60 mL culture flasks and incubated at 37 °C in a 5% CO_2_ atmosphere.

### 2.9. Human Peripheral Blood Mononuclear Cells (PBMCs) Isolation

PBMCs isolation was conducted following the protocol of Freshney [13] using Ficoll-Paque^TM^ Plus (Cytiva, Uppsala, Sweden). Sodium heparin anticoagulated venous blood (buffy coat) samples were obtained from the blood bank of the Srinagarind Hospital. The isolated PBMCs, with a viability of at least 98% as determined by hemacytometer counting following the protocol of Louis and Siegel [14], were used for toxicity testing. The cells were suspended in modified RPMI-1640 medium supplemented with L-glutamine, 10% FBS, 100 µg/mL streptomycin, and 100 U/mL penicillin, at a concentration of 1 × 10^6^ cells/mL, according to Arimilli et al. [12].

### 2.10. Cytotoxicity Testing via (3-(4,5-dimethylthiazol-2-yl)-2,5-diphenyltetrazolium bromide) Tetrazolium Reduction (MTT) Assay

The experiments were conducted on 96-well plates, with 100 µL of the prepared cell suspension added to each well, followed by 10 µL of the extract at eight different concentration levels. The plates were then incubated for 24 h at 37 °C in a 5% CO_2_ atmosphere. The MTT assay, IC_50_ and LD_50_ calculations were performed following the protocols outlined by Freshney [13] and Sudmoon et al. [15]. The hazardous levels of the compounds were categorized according to the guidelines provided by the World Health Organization [16].

### 2.11. Genotoxicity Testing via Comet Assay

The comet assay was conducted following the methods described by Sudmoon et al. [15] and Singh et al. [17]. All the extracts were tested for genotoxicity at their IC_50_ concentration, or at a maximum working concentration when no IC_50_ value was determined.

### 2.12. Programmed Cell Death Evaluation by Flowcytometry Assay

The cells were treated with a single working concentration following the previous mention of the comet assay. After the harvesting and washing steps, the cells were gently resuspended with 100 µL of 1× annexin-binding buffer. Subsequently, the resuspended cells were dyed with 5 µL of FITC Annexin V and 1 µL of the 100 µg/mL PI working solution (Invitrogen, ThermoFisher Scientific, Waltham, MA, USA); then, the cells were incubated at room temperature for 15 min. After the incubation period, the 400 µL of 1X annexin-binding buffer was added and mixed gently. The stained cells were analyzed by flow cytometer, measuring the fluorescence emission at 530 nm and >575 nm [18]. The data were plotted in two-dimensional dot plots between the stained cells with PI and FITC annexin V. The plots can be divided into four regions corresponding to (1) viable cells which are negative to both probes; (2) apoptotic cells which are PI negative and annexin positive; (3) late apoptotic cells which are PI and annexin positive; and (4) necrotic cells which are PI positive and annexin negative [19].

### 2.13. Cell Cycle Arrest by Flowcytometry Assay 

The harvested cells were fixed in 70% ethyl alcohol at 4 °C for 30 min without light, then the cells were washed twice with 1× PBS. After that, the 0.5 mL of PI/RNase staining solution (Invitrogen, ThermoFisher Scientific, Waltham, MA, USA) was added and incubated at 37 °C for 30 min in the dark. The cell cycle distribution was generated by flow cytometer using a 532 nm excitation with a 585/42 bandpass [20]. The G0/G1 and G2/M phase histogram peaks were separated by the S phase distribution.

### 2.14. Statistical Analysis 

All values were expressed as the mean ± standard deviation (S.D.). For statistical significance, the non-parametrical Kruskal–Wallis test was selected to compare data within a group containing a single variant. In addition, the non-parametrical Mann–Whitney U test was employed to test the difference in data between groups using GraphPad Prism version 8.0.2 (GraphPad Software, Boston, MA, USA). To express the confidence level, the definition of statistical significance was set at *p* < 0.05.

## 3. Results

### 3.1. Phytochemical Profiles Contained in the Three Cratoxylum Leaf Extracts

**Figure 1:**
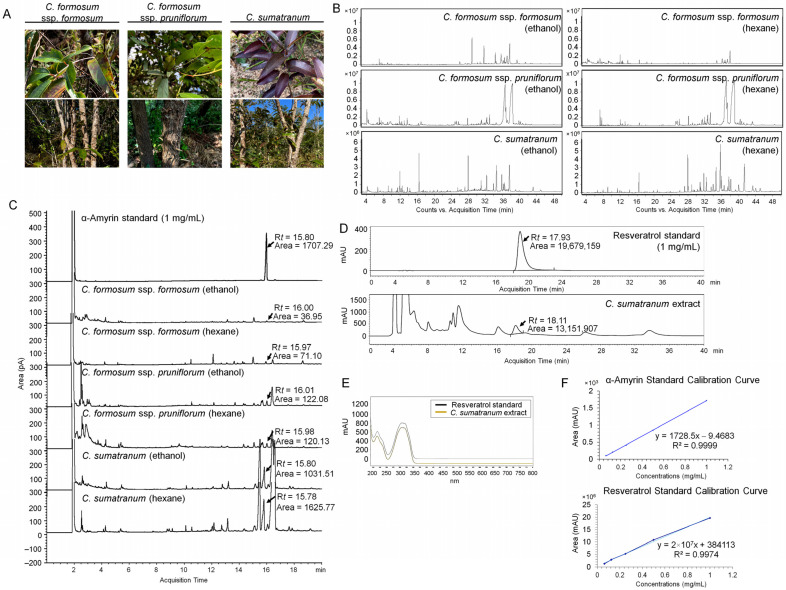
The phytochemical identification in the *Cratoxylum* extracts. (**A**) The two studied *Cratoxylum* species, and two subspecies; (**B**) GC-MS chromatogram of each extracted solvent and sample; (**C**) the GC chromatogram of α-amyrin detected in each extracted solvent and sample indicating Rt and areas; (**D**) the HPLC chromatogram of resveratrol in 80% ethanol extract of *C. sumatranum* leaf; (**E**) the spectrum comparison between the resveratrol standard and sample; (**F**) the standard calibration equations of α-amyrin and resveratrol.

The studied *Cratoxylum* species, namely *C. formosum* ssp. *formosum*, *C. formosum* ssp. *Pruniflorum*, and *C. sumatranum* have morphological characters, especially *C. sumatranum* which has a purplish red color on young leaves and green chlorophyll mixed with a purple color on mature leaves (Figure 1A). Aligned to the differences mentioned, the derived phytochemical constituents also varied in each species. The GC-MS chromatograms (Figure 1B) revealed plenty of phytochemical groups in the *Cratoxylum* species divided by the extracted solvents. There were three major substances found, namely α-amyrin, a pentacyclic triterpenoid which is a predominant bioactive compound detected in the *C. formosum* ssp. *formosum* ethanol extract, and both the ethanol and hexane extracts of *C. formosum* ssp. *pruniflorum* and *C. sumatranum*, found at high levels of 48.49% and 51.92%, respectively (Table 1). The second was lupeol, a pentacyclic triterpenoid which was detected in all *Cratoxylum* extracts except the *C. formosum* ssp. *pruniflorum* ethanol extract, and which was at its highest level of 49.79% in the *C. formosum* ssp. *formosum.* The third compound was caryophyllene, a sesquiterpene which was identified in all *Cratoxylum* ethanol and hexane extracts and had its highest level of 17.57% in the *C. formosum* ssp. *formosum* ethanol extract. Other key bioactive compounds, such as catechol, β-selinene, phytol, γ-tocopherol, stigmasterol, and γ-sitosterol, were likewise detected in the *Cratoxylum* leaf extracts (Table 1).

### 3.2. Quantitative Analysis of α-Amyrin and Resveratrol in the Cratoxylum Leaf Extracts 

For α-amyrin analysis in all studied samples, the ethanol and hexane *Cratoxylum* extracts were submitted to GC-FID. The retention time (R*t*) of the α-amyrin standard was detected at 15.80 min. The α-amyrin substance was detected in all studied *Cratoxylum* extracts with the Rt between 15.78 and 16.00 min giving the highest level on the chromatograms shown in Figure 1C, corresponding with the high percentages on the GC-MS results. The *C. sumatranum* in both extracted solvents had high amounts of α-amyrin of 0.024 mg/g of dried leaves for the ethanol extract and 0.04 mg/g of dried leaves for the hexane extract (Table 2). 

For the resveratrol analysis in the *C. sumatranum* extracts, the resveratrol, a stilbenoid, was found in the 80% ethanolic extract of *C. sumatranum* at the Rt of 18.11 min. The Rt of the standard was detected at 17.93 min. So, the spectrums of the standard and the samples were compared, and it was authenticated that the compounds with a different Rt were identical (Figure 1D,E). The resveratrol concentration and quantity in the mentioned species were 0.640 mg/mL and 0.064 mg/g dried leaf, respectively. The linear calibration equations of both standards plotted between concentration and area, providing the dependable correlation coefficient (R^2^) as 0.9999 for α-amyrin and 0.9974 for resveratrol (Figure 1F).

### 3.3. Cytotoxicity Evaluation of the Studied Cratoxylum Samples on the Cancer Cells and PBMCs

**Figure 2:**
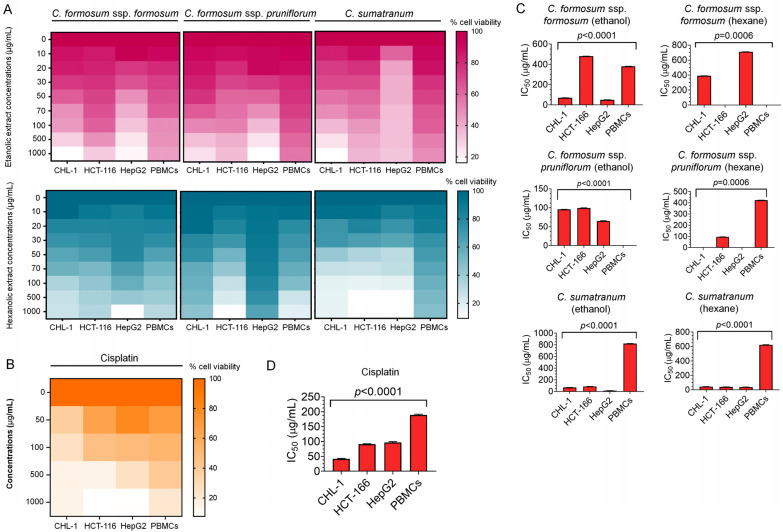
The cytotoxic effects of the *Cratoxylum* extracts. The heat map showing the cell viability capacity after treatment with the *Cratoxylum* working concentrations, 10, 20, 30, 50, 70, 100, 500, and 1000 µg/mL indicated by color to divide the (**A**) ethanol (in pink) and hexane (in blue) extracts; and (**B**) cisplatin, the dark color indicating high cell viability capacity, whereas the light color displays low cell viability capacity; (**C**) the IC_50_ values of each *Cratoxylum* working concentration; and (**D**) cisplatin showing the significant result (*p* < 0.05). The IC_50_ values were indeterminate in *C. formosum* ssp. *formosum* (hexane) and *C. formosum* ssp. *pruniflorum* (shown by the number >1000).

To evaluate the cytotoxicity levels of the ethanol and hexane *Cratoxylum* extracts, the eight working concentrations (10, 20, 30, 50, 70, 100, 500, and 1000 µg/mL) were applied to the three human cancer cell lines including CHL-1, HCT-116, and HepG2 as well as normal human immune cells PBMCs for 24 h (Figure 2A). The results disclosed that the highest working concentration of the *C. formosum* ssp. *formosum* ethanol extract showed lower percentages of cell viability of 17.77 ± 0.03 in CHL-1, 34.05 ± 0.09 in HCT-116, and 22.31 ± 0.05 in HepG2 than the hexane extract. Accordingly, the ethanol *C. formosum* ssp. *pruniflorum* at the highest working concentration alike revealed lower percentages of cell viability of 20.45 ± 0.07 in CHL-1, 30.87 ± 0.11 in HCT-116 and 10.94 ± 0.04 in HepG2 than the hexane extract (Figure 2A and Table 3). Furthermore, both of the ethanol and hexane extracts of *C. sumatranum* at the highest concentration revealed lower percentages of cell viability in CHL-1 as 16.24 ± 0.04 for the ethanol extract and 16.57 ± 0.01 for the hexane extract than the other extracts. Remarkably, the hexane *C. sumatranum* working concentration at the highest concentration provided the lowest percentage of cancer cell viability compared to other working concentrations of 9.75±0.00 in HCT-116 and 9.83 ± 0.01 in HepG2 (Figure 2A and Table 3). For the human PBMCs, the percentages of cell viability after being treated with the *Cratoxylum* working concentrations were higher than the cancer cells (Figure 2A and Table 3).

To compare the ability of the *Cratoxylum* extract for anti-cancer proliferation, cisplatin, a chemotherapeutic drug, was employed as a positive control. The percentages of cell viability at the highest concentration (1000 µg/mL) of cisplatin were 14.96 ± 0.01 in CHL-1, 7.83 ± 0.02 in HCT-116, 8.86 ± 0.01 in HepG2, and 22.16 ± 0.03 in PBMCs(Figure 2B and Table 3). The mentioned cell viability treated with cisplatin was lower than the *Cratoxylum* working concentrations. However, the IC_50_ values of each treatment were analyzed to indicate their efficiency revealing significant suppression of the cancer cell proliferation (Figure 2C). The lowest IC_50_ value of each sample and cells, 43.75 (CHL-1), 40 (HCT-116), 16 (HepG2), and 380 (PBMCs) µg/mL indicated the highest efficiency for anti-cancer activity compared within the *Cratoxylum* working concentration groups. They indicated that the hexane *C. sumatranum* working concentration had lower IC_50_ values in CHL-1 and HCT-116 at 43.75 and 40 µg/mL than the other working concentrations. Meanwhile, the ethanol *C. sumatranum* working concentration exhibited a lower IC_50_ value in the HepG2 at 16 µg/mL than the other working concentrations (Table 3). The IC_50_ values of cisplatin were higher than all the *Cratoxylum* working concentrations, except in the CHL-1 line (Figure 2D and Table 3). The IC_50_ values were used for LD_50_ calculation and extrapolated following WHO, 2009. Our extrapolated data on predicted LD_50_ doses of all *Cratoxylum* samples were classified as moderately hazardous class II for oral consumption, 50-100 mg/kg body weight of rats as a solid, 200-2000 mg/kg body weight of rats as a liquid, where liquids and solids refer to the physical state of the active ingredient being classified (Table 3).

### 3.4. Genotoxicity Effect of the Studied Cratoxylum Samples on the Studied Cells

**Figure 3:**
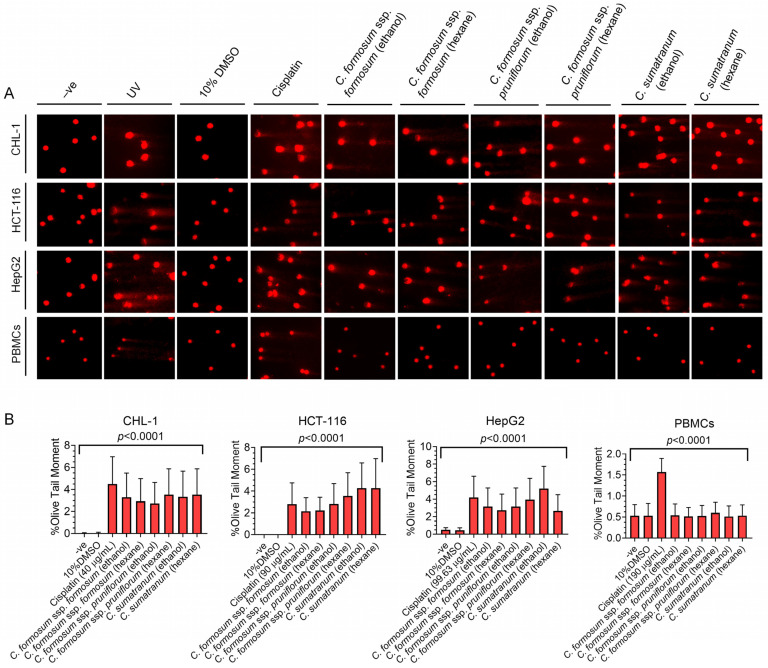
The genotoxicity evaluation of the *Cratoxylum* working concentration at IC_50_ or the highest concentrations shown in Table 3 compared to cisplatin acting as a chemo-drug at the determined concentration on the studied cells through comet assay image (200×). (**A**) The DNA damage fragment detected in cancer cells after treating with *Cratoxylum* extract and not detected in PBMCs. The untreated cell was employed as negative control (-ve), 10% DMSO was used as a vehicle control, treated UV cell acted as a positive control; (**B**) the OTM values of the cancer cells after treatment with the *Cratoxylum* working concentrations and cisplatin were higher than untreated cells but have no difference in PBMCs, showing the genotoxic effect on cancer cells.

From the cytotoxicity results, the concentration at the IC_50_ level or the highest concentration (when IC_50_ values were lacking) were used to evaluate the genotoxicity on cells for the corresponding experiment (Table 3). The results revealed that the determined concentrations of all *Cratoxylum* working concentrations as well as cisplatin had the effect of inducing significant DNA damage on cancer cells compared to untreated cells as a negative control (Table 4). The DNA fragments of cancer cells were detected after treatment with the *Cratoxylum* working concentrations (Figure 3A). Notably, the working concentrations did not show an effect on PBMC DNA damage, and the olive tail moment (OTM) values of each treatment were similar to the negative control (Figure 3A,B and Table 4).

### 3.5. Programed Cell Death Detection in Cancer Cells and Normal Immune Cells (PBMCs) for Efficacy Testing at the IC_50_ or the Highest Concentration Value of the Cratoxylum Samples

**Figure 4:**
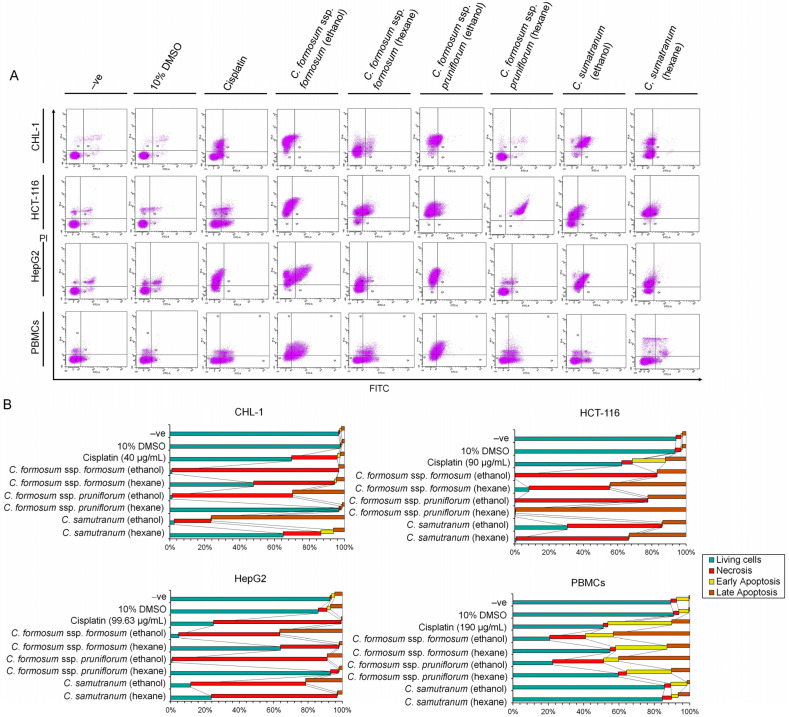
The programed cell death evaluation of the *Cratoxylum* extracts. (**A**) The two-dimensional dot plots of single cell plotting between PI and FITC to identify apoptotic, necrotic, and living cells; (**B**) the bar chart of each cell provided the percentage of each programed cell death compared to each experiment condition.

As an identical condition to the comet assay, the specifically determined concentration of the working concentration was further employed to study programed cell death. The derived results showed that all determined *Cratoxylum* working concentrations (Table 3) induced extreme apoptosis and necrosis on cancer cells and had an immediate effect on PBMCs (Figure 4A,B). Initially with the CHL-1 cell line, the *C. sumatranum* ethanolic extract showed an effect on apoptosis induction at 76.5%, as well as the cell being induced with ethanolic *C. formosum* ssp. *formosum* and *C. formosum* ssp. *pruniflorum* extracts to incur necrosis at 95.7% and 69% (Figure 4A,B). This was followed by the HCT-116 cell line, where the hexanolic *C. formosum* ssp. *pruniflorum* extract strongly induced apoptosis at 99.9%. The ethanolic extract of this species also showed an effect on necrosis induction of 77.6%. Additionally, the hexanolic *C. formosum* ssp. *formosum* working concentration had an effect on both programed cell death with the slightly different level of 44.7% for apoptosis and 47.1% for necrosis. However, the *C. sumatranum* working concentrations also showed an immediate effect on apoptosis and necrosis for this cell line (Figure 4A,B). For the HepG2 cell line, the ethanolic *C. formosum* ssp. *formosum* and both of the *C. sumatranum* working concentrations induced the cell apoptosis at 36.5% and 21.4%, as well as the ethanolic *C. formosum* ssp. *pruniflorum* and both of *C. sumatranum* working concentrations inducing necrosis on the HepG2 cell line at 90.4% (*C. formosum* ssp. *pruniflorum* ethanol), 66.9% (*C. sumatranum* ethanol), and 73.3% (*C. sumatranum* hexane) (Figure 4A,B).

From these results, almost all *Cratoxylum* working concentrations showed a high percentage on live PBMCs cells, except the ethanolic *C. formosum* ssp. *formosum* and both *C. formosum* ssp. *pruniflorum* extracts. Remarkably, the *C. sumatranum* working concentrations strongly induced the cancer cell to incur programmed cell death for anti-cancer proliferation activity, but did not affect PBMCs as normal immune cells. These working concentrations showed the high viability capacity of PBMCs of 84.5–85.7% (Figure 4A,B). Noticeably, the cisplatin in the concentration that was used to treat the corresponding cell showed low efficiency when compared to the *Cratoxylum* working concentrations (Figure 4B).

### 3.6. Cratoxylum Sample Activity Testing on Cell Cycle Distribution Using IC_50_ or the Highest Concentration Value

**Figure 5:**
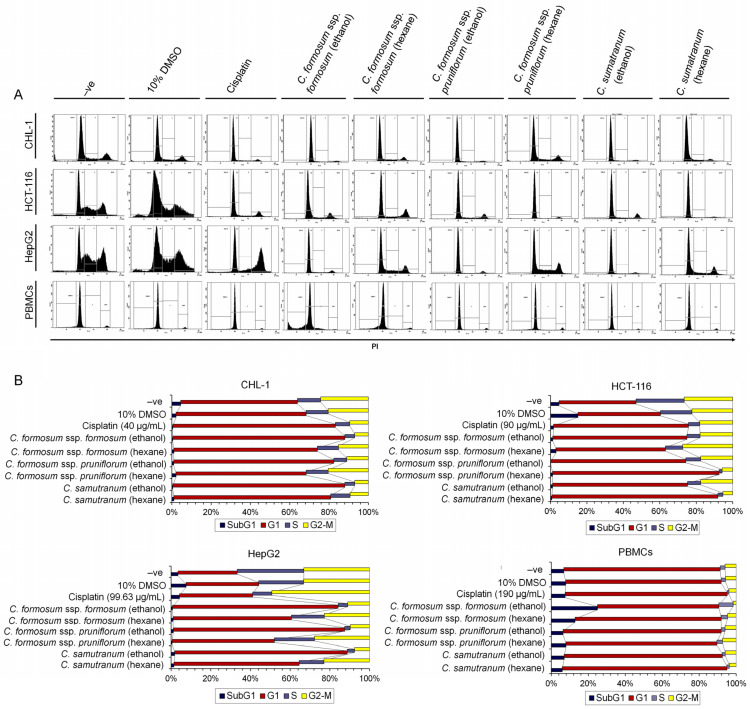
The cell cycle distribution analysis after treatment with the working concentrations shown in Table 3. (**A**) The histogram of cell cycle showing the DNA content peak in subG1, G1, S, and G2-M phases; (**B**) the bar chart of each cell line in cell cycle phases containing the percentages of DNA content in each experimental condition.

From the programmed cell death results, these next experiments were designed with identical conditions to evaluate the cell cycle distribution after treating with the determined working concentrations (Table 3). The results revealed that the *Cratoxylum* working concentrations arrested the cell cycle of cancer cells including CHL-1, HCT-116, and HepG2 within the G1 phase and also suppressed the cell to not continue to the S-phase as a DNA synthesis phase or the G2-M phase for cell division. Interestingly, the working concentrations did not affect PBMCs when compared to the untreated cell as a negative control. Furthermore, the *Cratoxylum* working concentrations displayed higher potency than cisplatin (Figure 5A,B).

## 4. Discussion

**Figure 6:**
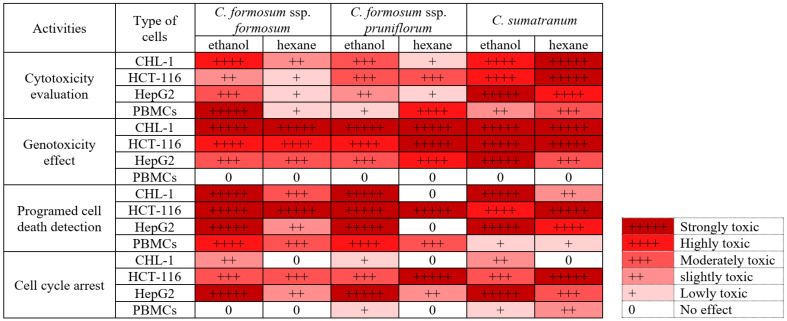
The summary of toxicity properties on cancer cells and PBMCs of the studied *Cratoxylum* species.

This research has had significant results in relation to two points: the first is the discovery of a new source of resveratrol that has unlimited quantities and many uses that are beneficial to humans. The second point is the results showing that the studied *Cratoxylum* samples have properties that are toxic to cancer cells, but that they have little toxicity to PBMCs confirmed by all factors studied, which are shown in Figure 6.

The study of the phytochemical composition of the leaf extracts of the two *Cratoxylum* species and two subspecies, including three taxa using GC-MS, detected many phytochemicals as shown in Table 1, but the three major compounds included α-amyrin, (one out of several amyrin isomers including β-amyrin, differing only on the position of the methyl group on the triterpene skeleton) which is a triterpenoid substance demonstrating potential anti proliferative, antinociceptive, antioxidant, and anti-inflammatory activities via antibacterial activity against *Propionibacterium acnes* [21,22,23,24]. The α-amyrin was detected in all samples of *Cratoxylum* leaf extracts and had the highest relative percentage content in *C. sumatranum* of 48.49–51.92%. After analyzing the amount of this substance using the GC-FID method, it was found that α-amyrin from *C. sumatranum* extracts had the highest amount at 0.024 mg/g-dried leaves for ethanol extract and 0.04–0.955 mg/g dried leaves (Table 2), which were consistent with the percentage relative content detected by GC-MS (Table 1).

The young shoots and young leaves showed a purplish red color in all the studied plant species, but no resveratrol was found using GC-MS investigation. However, the authors observed more characters than previously mentioned, including that *C. sumatranum* young plant has a dark purple color, and green chlorophyll mixed with a purple color on mature leaves, which, when dried, have a fully purple color. One notable clue is the resveratrol found in the skins of red grapes [25]. So, the *C. sumatranum* leaves were investigated for resveratrol using HPLC. The results of the study were as expected, with very high levels of resveratrol found, 0.640 mg/mL and 0.064 mg/g in the dried leaves (Table 2). This is the first such discovery in plant leaves, which are easy to obtain in large amounts, a discovery that was guided by the plants’ dark purple leaves, and it can be assumed that resveratrol is probably related to the pigment of the plant leaves. The researchers suspect that other species in the *Cratoxylum* genus are likely to have resveratrol as well, especially in young leaves. These species should be studied in the future for identification of potential unlimited human benefit. This is because resveratrol, a natural polyphenolic non-flavonoid antioxidant, is a phytoalexin and has important effects with human health benefits such as possessing antioxidants, being cardioprotective, and having anticarcinogenic and antitumor properties. The previous quantity found was not very high and was within the small study unit including grapes, berries, cocoa, and nuts. It is synthesized by plants as a defense against parasites such as bacteria or fungi [26]. The *C. sumatranum* leaves do not seem to synthesize it as a defense agent, but rather for its natural contribution of the purple color in the plant leaves. Therefore, this solved the problem of obtaining large amounts of resveratrol as the leaves are a large plant organ, and best of all, the resveratrol can continue to be created after the leaves fall off in the dry season. It can benefit human life in unlimited ways, through both traditional methods and via modification into various products such as cosmetics, future foods in the form of functional foods, nutraceutical foods, novel foods, etc. In addition, many important substances were detected (Table 1) in *Cratoxylum* leaf extracts, including lupeol, which is an anti-cancer and anti-inflammatory agent [27]; caryophyllene, that has several biological activities such as being antibacterial, antioxidant, gastroprotective, anxiolytic, anti-inflammatory, anti-aging, assisting in re-epithelialization, and with neuroprotective potential in animal models [28,29]; catechol, an antioxidant revealing itself to be a strong reducing reactive oxygen species agent [30] that has several valuable properties including anti-viral, antibacterial, anti-aging, and hypotensive effects [31], and which is also a component of the plants’ natural products that reduces cholesterol and inhibits lipase activity leading to an innovation for weight loss and high cholesterol treatments without any side effects [32]; β-selinene, a natural product that is found in essential oils from a variety of plants providing a functional antioxidant with antiradical and antimicrobial properties [33]; phytol, which has antinociceptive, antioxidant [34], and anti-inflammatory activities [35], and is a chlorophyll component that produces anti-hyperalgesia, anti-inflammatory, and antiarthritic effects [36]; γ-tocopherol, which shows beneficial effects in Alzheimer’s disease, is a form of vitamin E which acts as an antioxidant and reduces the level of reactive oxygen species [37]; stigmasterol, one of the most common plant sterols found in a variety of natural sources, including vegetable fats or oils from many plant, currently being examined for its various biological activities such as anti-cancer, anti-osteoarthritis, anti-inflammatory, antidiabetic, immunomodulatory, antiparasitic, antifungal, antibacterial, antioxidant, and neuroprotective properties [38]; and γ-sitosterol, an epimer of *β*-sitosterol, has been found to possess antihyperglycemic activity by increasing insulin secretion in response to glucose against cancer through the growth inhibition and cell cycle arrest on the apoptosis of cancer cells, against colon and liver cancer cell lines [39]. The chemical composition of the studied *Cratoxylum* sample species, including the major and minor substances contained, supports various *Cratoxylum* activities given the substance components’ quantities and function, providing many benefits of this genus that can be used to improve to human health [9]. According to the criteria of a multicomponent drug, this Chinese natural medicine has a poly-pharmacological effect naturally based on the ‘multi-component, multi-target and multi-pathway’ principle, alleviating several ailments simultaneously [40,41,42].

Even though members of the *Cratoxylum* species are edible, they should be tested for toxicity to determine the extent or amount for safe consumption, especially after processing. In addition, they are also tested for their uses in both food and disease treatment. So, toxicity assessment is an important process, and we found that the cytotoxicity testing of the studied *Cratoxylum* samples showed very striking results. All *Cratoxylum* samples showed effective biological activity, significantly inhibiting the growth of the three cancer cell lines: CHL-1, HCT-116, and HepG2. In particular, *C. sumatranum* leaf extracts showed lower percentage survival and IC_50_ values against all cancer cell lines than the other two *Cratoxylum* subspecies and higher efficiency than cisplatin, a modern chemotherapeutic drug which is currently being used, confirmed by the IC_50_ values (Figure 2D and Table 3). The results with PBMCs are also interesting since at the highest concentration level of 1000 µg/mL, all the studied *Cratoxylum* extracts showed a higher percentage of survival of PBMCs which are cells in the normal human immune system (Figure 2 and Table 3). Despite this, the *Cratoxylum* extracts are classified as moderately hazardous class II by WHO [14], where oral consumption of 50–100 mg/kg/body weight of solid or 200–2000 mg/kg/body weight of liquid will exceed the toxicity level when this amount is given to rats which have completely different genetic makeup than humans. Therefore, the hazardous class is just an indicator of consumer concentrations. In-depth, toxicity testing in genetic materials showed that the concentration level at IC_50_ or the highest concentration level of *Cratoxylum* leaf extracts did not induce DNA breaks in PBMCs, but it did significantly cause DNA breaks in cancer cells (Figure 3 and Figure 6, Table 4).

Moreover, at these concentration levels (used in the comet assay), the extracts were able to induce the death of all cancer cell lines by both apoptosis and necrosis (Figure 4), as well as significantly decrease the DNA quantity in the S and G2-M phases (Figure 5), revealing that the studied *Cratoxylum* extracts have the effect of blocking the growth process of the cancer cell lines. The PBMC results showed a high percentage cell viability of 84.5–85.7%, except for the ethanolic extract working concentrations of the two subspecies of *C. formosum* that induced PBMC death by apoptosis and necrosis and showed low cell viability percentages at 20.8–22.4%. These data confirmed that the three studied *Cratoxylum* samples are very specific at destroying cancer cells which follow from the substances they contain such as resveratrol and α-amyrin, which have anticancer and anti-inflammatory functions [21,22,23,24,25].

## 5. Conclusions

In conclusion, we made the first finding of resveratrol in the leaves of *C. sumatranum* and identified that all *Cratoxylum* extracts are toxic to cancer cell lines but are only slightly toxic to PBMCs at the cell and are not toxic to DNA levels; all sample extracts induced both apoptosis and necrosis in cancer cell lines, and the DNA quantity in the S and G2-M phases significantly decreased. The *C. sumatranum* extracts did not induce apoptosis and necrosis in PBMCs, showed a high percentage cell viability, but the ethanolic extracts of the two subspecies, *C. formosum,* induced PBMC apoptosis and necrosis. These data confirmed that the three studied *Cratoxylum* samples have an inhibitory effect on the growth of cancer cells and show low toxicity to PBMCs. *C. sumatranum* leaf extracts showed better biological activity than the other *C. formosum* leaf extracts, which may be due to the combined activity of substances such as α-amyrin and resveratrol. All studied samples are deserving of more experiments, with clinical trials for further application to plant-derived products, especially *C. sumatranum*.

## Figures and Tables

**Table 1:** Phytochemical profiles contained in the three *Cratoxylum* leaf extracts detected by GC-MS.

R*t* (min)	Phytochemical Name	Molecular Formula	% Relative Content					
			*C. formosum* ssp. *formosum*		*C. formosum* ssp. *pruniflorum*		*C. sumatranum*	
			Ethanol	Hexane	Ethanol	Hexane	Ethanol	Hexane
4.28	Catechol	C_6_H_6_O_2_	-	-	12.57	-	3.86	-
4.63	5-Hydroxymethylfurfural	C_6_H_6_O_3_	-	-	2.2	-	0.38	-
6.73	Tetracosane	C_24_H_50_	-	12.9	-	-	-	-
7.18	Caryophyllene	C_15_H_24_	17.57	0.9	3.68	1.83	0.66	0.93
7.29	α-Bergamotene	C_15_H_24_	-	-	-	-	-	0.05
7.42	Geranyl acetone	C_13_H_22_O	-	-	-	-	-	0.06
7.60	Humulene	C_15_H_24_	0.6	-	1.16	0.56	0.31	0.42
7.66	α-Farnesene	C_15_H_24_	1.12	-	-	-	-	-
7.85	α-Cubebene	C_15_H_24_	1.41	-	-	-	-	-
7.99	β-Selinene	C_15_H_24_	4.38	-	-	-	-	-
8.22	α-Curcumene	C_15_H_22_	1.19	-	-	-	-	0.05
8.73	Benzoic acid, 4-ethoxy-, ethyl ester	C_11_H_14_O_3_	1.09	0.66	-	-	-	-
8.79	trans-Nerolidol	C_15_H_26_O	-	-	-	-	-	0.07
9.17	Hexadecane	C_16_H_34_	-	-	1.36	-	0.16	-
9.18	Caryophyllene oxide	C_15_H_24_O	2.17	0.41	-	0.43	-	-
10.98	Tetradecanoic acid	C_14_H_28_O_2_	-	-	-	-	0.1	-
11.40	Octadecane	C_18_H_38_	-	-	0.76	-	0.11	-
11.91	Phytol, acetate	C_22_H_42_O_2_	1.19	-	6.13	0.36	0.91	0.49
12.35	3,7,11,15-Tetramethyl-2-hexadecen-1-ol	C_20_H_40_O	0.51	-	3.65	-	0.51	0.22
12.60	4,4,8-Trimethyltricyclo [6.3.1.0(1,5)]dodecane-2,9-diol	C_15_H_26_O_2_	-	-	0.98	-	-	-
13.04	Farnesyl acetone	C_18_H_30_O	-	-	-	-	0.36	0.3
13.75	n-Hexadecanoic acid	C_16_H_32_O_2_	0.55	-	4.51	-	1.4	0.15
14.24	Hexadecanoic acid, ethyl ester	C_18_H_36_O_2_	-	-	0.65	-	0.11	-
14.83	β-Elemene	C_15_H_24_	0.83	-	-	-	-	-
16.42	Phytol	C_20_H_40_O	2.53		3.44	0.27	0.17	0.21
17.00	Linolenic acid	C_18_H_30_O_2_	-	-	-	-	0.49	-
17.34	Octadecanoic acid	C_18_H_36_O_2_	-	-	-	-	0.09	-
17.43	Linolenic acid, ethyl ester	C_20_H_34_O_2_	-	-	-	-	0.09	-
18.21	Trolox	C_14_H_18_O_4_	-	-	2.2	-		-
23.17	Glycerol β-palmitate	C_19_H_38_O_4_	-	-	0.56	-	0.08	-
25.25	5a,9,9-Trimethyloctahydro-2H,4H-cyclopropa[e][3] benzoxepine-2,4-dione	C_14_H_20_O_3_	-	-	-	-	2.07	2.27
26.09	Heptacosane	C_27_H_56_	-	7.87	-	1.24		
27.95	Squalene	C_30_H_50_	0.82	-	0.68	0.35	0.72	0.78
28.92	Nonacosane	C_29_H_60_	-	3.45	-	15.5		0.22
31.04	ᵞ-Tocopherol	C_28_H_48_O_2_	-	6.06	-	-	0.24	0.23
31.75	Hentriacontane	C_31_H_64_	-	1.01	-	12.54	-	0.84
31.98	Cycloartenol acetate	C_32_H_52_O_2_	-	-	-	-	0.33	-
32.36	dl-α-Tocopherol	C_29_H_50_O_2_	2.46	-	-	-	0.74	0.67
32.59	2,6-Bis(3,4-methylenedioxyphenyl)-3,7-dioxabicyclo(3.3.0)octane	C_20_H_18_O_6_	-	-	-	-	0.5	-
33.07	Serratene	C_30_H_50_	-	-	-	-	1.9	2.01
34.44	1,30-Triacontanediol	C_30_H_62_O_2_	-	-	-	8.93	-	-
34.61	Stigmasterol	C_29_H_48_O	-	-	5.12	1.66	0.47	0.53
35.83	ᵞ-Sitosterol	C_29_H_50_O	2.18	-	8.33	5.98	-	-
36.67	Lupeol	C_30_H_50_O	49.79	21.28	-	2.52	28.58	30.88
36.86	β-Amyrin	C_30_H_50_O	-	-	-	5.48	4.52	4.71
37.16	Lupenone	C_30_H_48_O	-	-	6.99	8.94	-	-
38.20	α-Amyrin	C_30_H_50_O	8.29	-	33.33	33.42	48.49	51.92
40.04	Bauerenol	C_30_H_50_O	-	-	-	-	0.9	1.16
40.89	Friedelanol	C_30_H_52_O	-	-	-	-	0.34	0.54
41.62	Friedelan-3-one	C_30_H_50_O	-	-	-	-	0.27	0.29

**Table 2:** Quantitative analysis of α-amyrin and resveratrol in the three *Cratoxylum* leaf extracts.

Extracted Solvents	*C. formosum* ssp. *formosum*		*C. formosum* ssp. *pruniflorum*		*C. sumatranum*	
	Concentration (mg/mL)	Quantity (mg/g-Dried Leaf)	Concentration (mg/mL)	Quantity (mg/g-Dried Leaf)	Concentration (mg/mL)	Quantity (mg/g Dried Leaf)
α-Amyrin						
Ethanol	0.027	0.001	0.076	0.003	0.601	0.024
Hexane	0.046	0.002	0.075	0.003	0.955	0.038
Resveratrol						
80% Ethanol	-	-	-	-	0.640	0.064

**Table 3:** The percentages of cell viability, IC_50_ values, and LD_50_ values of each cell line after treating with the *Cratoxylum* working concentrations and cisplatin.

Experiments	% Cell Viability (mean ± SD)						
	Cisplatin	*C. formosum* ssp. *formosum*		*C. formosum* ssp. *pruniflorum*		*C. sumatranum*	
		Ethanol	Hexane	Ethanol	Hexane	Ethanol	Hexane
CHL-1	14.96 ± 0.01–37.18 ± 0.02	17.77 ± 0.03–88.51 ± 0.10	47.80 ± 0.06–99.77 ± 0.08	20.45 ± 0.07–90.91 ± 0.20	60.97 ± 0.08–97.83 ± 0.16	16.24 ± 0.04–83.50 ± 0.07	16.57 ± 0.01–94.36 ± 0.07
IC_50_ (µg/mL)	40	70	390	95	>1000	70	43.75
LD_50_ (µg/50Kg rat)	416.84	513.31	972.47	575.06	-	513.31	430.97
HCT-116	7.83 ± 0.02–64.31 ± 0.24	34.05 ± 0.09–85.56 ± 0.13	53.07 ± 0.07–92.83 ± 0.17	30.87 ± 0.11–89.05 ± 0.09	35.58 ± 0.11–92.32 ± 0.13	37.01 ± 0.21–89.23 ± 0.13	9.75 ± 0.00–93.93 ± 0.12
IC_50_ (µg/mL)	90	480	>1000	98	96	87	40
LD_50_ (µg/50Kg rat)	563.61	1050.56	-	581.75	577.30	556.55	416.84
HepG2	8.86 ± 0.01–80.10 ± 0.04	22.31 ± 0.05–97.89 ± 0.11	36.73 ± 0.13–89.56 ± 0.10	10.94 ± 0.04–91.75 ± 0.11	74.05 ± 0.06–94.87 ± 0.23	32.28 ± 0.05–61.040.05	9.83 ± 0.01–85.65 ± 0.08
IC_50_ (µg/mL)	99.63	50	710	63.50	>1000	16	39
LD_50_ (µg/50Kg rat)	585.33	452.92	1215.26	495.03	-	296.44	412.93
PBMCs	22.16 ± 0.03–68.27 ± 0.11	45.92 ± 0.01–91.81 ± 0.01	52.26 ± 0.01–93.94 ± 0.01	53.27 ± 0.00–94.49 ± 0.00	41.79 ± 0.02–95.96 ± 0.02	48.70 ± 0.03–91.82 ± 0.02	46.71 ± 0.04–89.96 ± 0.10
IC_50_ (µg/mL)	190	380	>1000	>1000	423	820	620
LD_50_ (µg/50Kg rat)	744.21	963.12	-	-	1002.30	1282.15	1155.50

**Table 4:** The OTM values of each cell after treatment with the corresponding extract.

Experiments	OTM Values (mean ± SD)								
	-ve	10% DMSO	Cisplatin	*C. formosum* ssp. *formosum*		*C. formosum* ssp. *pruniflorum*		*C. sumatranum*	
				Ethanol	Hexane	Ethanol	Hexane	Ethanol	Hexane
CHL-1	0.0414 ± 0.03	0.0399 ± 0.08	4.4950 ± 2.48	3.2875 ± 2.21	2.9309 ± 2.05	2.7148 ± 1.93	3.5335 ± 2.34	3.3423 ± 2.32	3.1684 ± 2.27
*p*-values	-	0.1929	<0.0001	<0.0001	<0.0001	<0.0001	<0.0001	<0.0001	<0.0001
HCT-116	0.0016 ± 0.00	0.0018 ± 0.01	2.7995 ± 1.96	2.1438 ± 1.25	2.2089 ± 1.23	2.8119 ± 1.88	3.5543 ± 2.13	4.2624 ± 2.32	4.2671 ± 2.71
*p*-values	-	0.6437	<0.0001	<0.0001	<0.0001	<0.0001	<0.0001	<0.0001	<0.0001
HepG2	0.4954 ± 0.25	0.4540 ± 0.28	4.1957 ± 2.42	3.1422 ± 2.14	2.7325 ± 1.85	3.1656 ± 2.12	3.9542 ± 2.42	5.2199 ± 2.53	2.6430 ± 1.87
*p*-values	-	0.1426	<0.0001	<0.0001	<0.0001	<0.0001	<0.0001	<0.0001	<0.0001
PBMCs	0.5327 ± 0.26	0.5314 ± 0.29	1.5705 ± 0.32	0.5150 ± 0.21	0.5401 ± 0.27	0.5255 ± 0.25	0.6009 ± 0.25	0.5141 ± 0.25	0.5338 ± 0.26
*p*-values	-	0.9591	<0.0001	0.7468	0.4350	0.7218	0.0247	0.4834	0.9819

## Data Availability

All data are provided in the main text.
